# Reply to “High energy and materials requirement for direct air capture calls for further analysis and R&D”

**DOI:** 10.1038/s41467-020-17204-6

**Published:** 2020-07-03

**Authors:** Giulia Realmonte, Laurent Drouet, Ajay Gambhir, James Glynn, Adam Hawkes, Alexandre C. Köberle, Massimo Tavoni

**Affiliations:** 1 RFF-CMCC European Institute on Economics and the Environment (EIEE), Centro Euro-Mediterraneo sui Cambiamenti Climatici, Milan, 20144 Italy; 2 Imperial College London, Grantham Institute, London, SW7 2AZ UK; 3 MaREI Centre, Environmental Research Institute, University College Cork, Cork, T23 XE10 Ireland; 4 Politecnico di Milano, Department of Management, Economics and Industrial Engineering, Milan, 20156 Italy

**Keywords:** Climate-change mitigation, Energy and society

**Replying to** S. Chatterjee *Nature Communications* 10.1038/s41467-020-17203-7 (2020)

We are writing in response to Chatterjee and Huang’s analysis on the material and energy requirements of Direct Air Carbon Capture and Sequestration (DACCS) plants, which cites our recently published paper on the potential role of such technologies in deep mitigation pathways. We agree with the authors that not only a thorough technoeconomic analysis but also a material and energy-needs assessment should be undertaken before any realistic role for DACCS can be finalized. This requires including life-cycle assessment (LCA) in the technoeconomic analysis provided by integrated assessment model (IAM) scenarios, going significantly beyond what is done in our analysis, and requiring major modeling innovation^1^, specifically to link chemical manufacturing sectors to energy and mitigation technology sectors, to a degree not done in IAMs so far.

Chatterjee and Huang’s calculations into the material and energy requirements to manufacture DACCS sorbents look reasonable in light of the assumptions that they make, and highlight in particular how energy-intensive DACCS sorbent manufacture might be.

Specifically, the additional energy requirement to manufacture what we term DAC1 technologies (those using a strong base liquid sorbent) results in a 37% uplift in energy demand compared with considering the operational energy alone. This could result in less cost-effective deployment than we calculated, although it should be noted that operational energy-efficiency improvements, innovation in sorbent manufacture, and lower sorbent-replacement rates than we assumed could conceivably mean that the total energy requirement of DAC1 is of the same order as in our calculations.

For the technology that we term DAC2 (using solid amine sorbents), the authors’ calculations suggest the energy required for sorbent manufacture is almost five times as high as that required for operating the technology, on the basis that the sorbent is monoethanolamine (MEA). We agree with the authors that this would make DAC2 unlikely to be able to deliver multi-Gt scale CO_2_ removals. We note, however, that there is still a high degree of confidentiality around the precise nature of the amine sorbents used in pre-commercial plants (such as those of Climeworks), so we would caution against basing any analysis solely on the MEA sorbents which the authors use as a reference. Nevertheless, their analysis proves very useful in setting out the scale of the challenge for any innovation in sorbents relative to MEA.

Three further key issues from our analysis should also be considered:

1. As already shown in our sensitivity analysis, energy requirements have a relatively limited influence on the deployment of DACCS, which is primarily governed by the rate at which DACCS is allowed to scale up (Figure 4a in our paper).
2. At the level of deployment shown in our paper, and given the decadal timescales involved, technological change could certainly play a big role in reducing the energy requirements of DACCS, which is not fully accounted for in our assessment.
3. The energy usage of mass deployment of DACCS is very significant even under our own assumptions, and this was one of the major messages of our paper. However, from a purely economic point of view (which is what matters most in a technoeconomic evaluation such as ours), the impact of the extra energy usage can be justified in light of the costs and availability of other mitigation and emissions removal options, which is accounted for in a whole energy systems analysis such as ours. In addition, in the WITCH model, the price of energy (both natural gas for heat and low-carbon electricity) is lower in the low-carbon scenarios where DACCS plays a significant role, compared with no climate policy scenarios, which would limit the economic impact of the energy costs to some extent.

As implied by Chatterjee and Huang, but also in our article, it would be unwise to utilize massive amounts of energy into the production and deployment of DACCS while that energy is still carbon-intensive. Nevertheless, the arguments above show that our modeling results remain valid irrespective of the engineering arguments put forward by Chatterjee and Huang. As a technology group and mitigation strategy, DACCS should still be actively considered for both a long-term and potentially major pre-2050 role, rather than written off as a financially and energetically costly distraction in the near-term. We cannot agree more with Chatterjee and Huang that further analysis, and further R&D into DACCS, is to be recommended.

## Data Availability

The data that support the findings of this study are available from the authors on request.
